# Supplementary material for: shRNA-Targeting Caspase-3 Inhibits Cell Detachment Induced by Pemphigus Vulgaris Autoantibodies in HaCaT Cells
Source: Int J Mol Sci. 2024 Aug 14;25(16):8864. doi: 10.3390/ijms25168864 (PMC11354573; doi:10.3390/ijms25168864)
Supplement: Supplementary file 1 [file ijms-25-08864-s001.zip › ijms-3094589-supplementary.pdf]

a b c

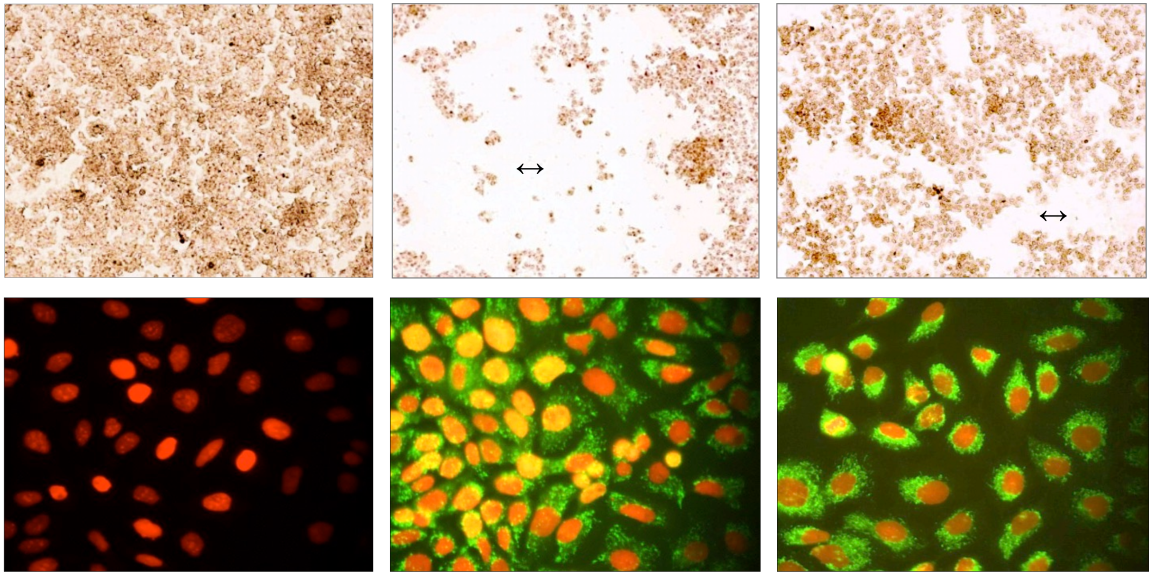

d e f

Supplementary Figures. Effect on *HaCaT* cells mediated by PV-IgG. Panel A and D: Cells treated with NHS-IgG (1 mg/mL), red fluorescence in nuclei means cells without apoptotic membranes B and E cells treated with camptothecin 4 µg/mL. Green fluorescence showing cells with phosphatidylserine exposure, similar effects were observed in cells treated with PV-IgG (1 mg/mL C and F). This assay was made by triplicate at 6 hours of incubation with each treatment. (a,b,c: bright field microscopy, 10X; d, Ee, Ff: fluorescence microscopy, 40X).
